# Primary Care of the Person Living with HIV

**DOI:** 10.3390/pathogens11040380

**Published:** 2022-03-22

**Authors:** Mary J. Kasten

**Affiliations:** Division of Public Health, Infectious Diseases and Occupational Medicine, Mayo Clinic, Rochester, MN 55901, USA; mkasten@mayo.edu

**Keywords:** HIV, primary care, communication, prophylaxis, antiretroviral therapy

## Abstract

Life-changing progress has been made over the past 30 years in the treatment of HIV infection. HIV has transformed from an illness that resulted in one complication after another and nearly always resulted in death to a chronic illness that for most patients is more easily managed than diabetes or heart disease. Antiretroviral therapy (ART) is now simple and well-tolerated. The most important priority of HIV treatment is ensuring that people living with HIV stay on continuous, effective ART. ART, although not curative, suppresses the virus and allows the immune system to recover. Even when the CD4 count remains low, suppressive ART helps prevent opportunistic infections and other HIV related complications. (1) Suppressive ART is important not only to the individual living with HIV health but is an important public health goal since people living with HIV will not transmit HIV to their sexual partners if their viral load is undetectable. (2) A respectful, culturally appropriate patient–provider relationship is one of the most important factors in keeping people living with HIV engaged in care. (3) Persons living with HIV deserve both excellent HIV and primary care. Some communities have providers that are experts in both, but often people living with HIV receive the best care by collaboration between their primary care provider and an HIV expert. This article is written to help primary care givers who are not HIV experts provide appropriate primary care to their patients who are living with HIV and emphasizes issues that deserve additional attention in people living with HIV compared to the general population

Life-changing progress has been made over the past 30 years in the treatment of HIV infection. HIV has transformed from an illness that resulted in one complication after another and nearly always resulted in death to a chronic illness that for most patients is more easily managed than diabetes or heart disease. Antiretroviral therapy (ART) is now simple and well-tolerated. The most important priority of HIV treatment is ensuring that people living with HIV stay on continuous, effective ART. ART, although not curative, suppresses the virus and allows the immune system to recover. Even when the CD4 count remains low, suppressive ART helps prevent opportunistic infections and other HIV related complications [1]. Suppressive ART is important not only to the health of the individual living with HIV but is an important public health goal since people living with HIV will not transmit HIV to their sexual partners if their viral load is undetectable [2]. A respectful, culturally appropriate patient–provider relationship is one of the most important factors in keeping people living with HIV engaged in care [3]. Persons living with HIV deserve both excellent HIV and primary care. Some communities have providers that are experts in both, but often people living with HIV receive the best care by collaboration between their primary care provider and an HIV expert. This article is written to help primary care givers who are not HIV experts provide appropriate primary care to their patients who are living with HIV and emphasizes issues that deserve additional attention in people living with HIV compared to the general population.

Establishing a high-quality, respectful relationship when caring for people living with HIV starts with non-judgmental and gender affirming communications. Ensuring preferred names and pronouns are used is part of the foundation for a high-quality patient–provider relationship. Understanding many sensitive life-style choices, which providers are often uncomfortable discussing with patients, is critical to providing high-quality primary care. This includes understanding the person’s sexual and drug use behavior in order to know best how to offer appropriate risk reduction counseling to reduce the risk both of acquiring other sexually transmitted infections (STIs) and blood borne diseases and how to eliminate the risk of HIV transmission to others. This information needs to be obtained in a non-judgmental manner with an explanation of why the information is important. The use of the communication technique legitimization is often helpful. A simple example of legitimization: “Many of my patients are sexually active with multiple partners, can you tell me who you have been sexually active with in the past year?”

Primary care clinicians should be familiar with HIV prevention including the use of condoms and both pre-exposure and post-exposure prophylaxis (PrEP and PEP) for HIV. Readers are referred to the respective Center for Disease Control (CDC) guidelines for further information [4,5]. Primary care clinicians need to understand that HIV-related stigma remains common and is a critical part of fighting the HIV pandemic in the US. HIV-related stigma discourages stigmatized people from obtaining HIV prevention, treatment, and care [6]. Primary care clinicians should avoid stigmatizing language and use preferred words and phrases. A simple example is avoiding the use of HIV patient and instead using person living with HIV to emphasize that the person is not in a constant state of illness and outside of a clinical context a person is not a patient. The CDC at hiv.gov has additional recommendations regarding preferred language [7].

Early diagnosis of HIV infection with prompt initiation of HIV treatment is crucial towards keeping people living with HIV healthy. Primary care clinicians should routinely discuss and be comfortable counseling their patients about HIV and other STI prevention. Screening for HIV is recommended for all pregnant persons and people between ages 15–65 by the United States Preventative Task Force (USPTF) at least once and for people at increased risk on a more frequent basis [8]. People at increased risk for acquiring HIV include men who have sex with men, people who inject drugs, commercial sex workers, and people whose sexual partner is living with HIV [3]. The American Geriatric Society also recommends screening older individuals, particularly as both the incidence and prevalence of HIV increases in the elderly [9]. Patients on PrEP for HIV need to be screened every 2–3 months [4]. Early diagnosis of HIV infection is advantageous to the individual in decreasing the likelihood they will experience HIV-related complications and benefits society. Many people, once they are diagnosed with HIV infection, change their behavior in an attempt to not transmit HIV to others [10]. Additionally, suppressive ART will ensure that HIV transmission through sex does not occur. Suppressive ART is therefore an important piece of HIV prevention efforts and has led to the public health slogan U = U or Undetectable equals Untransmissible. Multiple studies have shown that people living with HIV who are consistently undetectable on ART with no virus being found in their blood do not transmit HIV to their sexual partners [2,11]. The preferred screening test for people potentially living with undiagnosed chronic HIV infection is a fourth generation test combining serology and p24 antigen testing followed by automatic confirmatory testing if positive [12]. Testing for acute HIV infection requires both a serological test and a measure of HIV RNA (viral load) since serology can be negative early in infection. The HIV viral RNA (viral load) is expected to be very high (>100,000 copies/mL) in symptomatic acute HIV. The CDC has excellent guidelines for the diagnosis of HIV infection [12]. See Table 1 and Table 2 for clues regarding chronic and acute HIV infection with the caveat that many patients present atypically, and HIV infection should be considered in all persons presenting with an illness that has defied diagnosis [13,14].

The first visit with a person newly diagnosed with HIV infection is often primarily a counseling and educational visit. It is important that the patient has a good understanding of HIV and understands that it is a serious but very treatable infection. Many patients have a great sense of relief once the concept of U = U is explained to them. This, for many, is an important motivator that helps them stay compliant with treatment and allows them to live their life to the fullest. Some patients have an excellent understanding of HIV infection and are ready to start ART at the time of their first visit. Other patients need support and significant education about HIV and treatment options before they are ready to start treatment. Important goals of the first visit are to ensure the person living with HIV understands how to prevent the spread of HIV to others and that the person has the support and resources necessary to adjust to this serious diagnosis. A prompt follow-up visit with a partner, friend, or close family can also be very helpful in ensuring the person stays engaged in care. The primary care provider of people living with HIV needs to be aware that HIV stigma and discrimination remain prevalent. According to the CDC, nearly 8 out of 10 adults in the US receiving HIV care report feeling internalized HIV-related stigma. Correcting myths and stereotypes that the person living with HIV or their friends or family may have is an important role of all health care givers [7].

The initial evaluation of a person living with HIV is very comprehensive and often requires more than one visit to achieve. Ideally a multi-disciplinary team often including but not limited to a nurse, pharmacist, social worker, and the provider working together is best able to ensure that all the important information is obtained and documented, and that the person stays engaged in care.

The initial evaluation should include HIV-specific history including risk factors for HIV acquisition, likely route of acquisition, information on sexual partners including if their HIV status is known, use of PEP and or PrEP. The approximate dates of previous HIV testing, of their HIV diagnosis, HIV exposures, and symptoms consistent with acute HIV infection may allow the provider to estimate the date of HIV acquisition. A comprehensive history should be documented that includes the patient’s past medical history, psychiatric history, illnesses suggestive of opportunistic infections, surgical history, and allergies. Medications, including over the counter medications, vitamins and supplements, and any gender affirming hormonal treatment should be documented. A detailed review of systems looking for clues to HIV-related conditions and a detailed social history are important. The social history should include a discussion of the patient’s current and past sexual history, recreational drug use history, tobacco and alcohol use, occupational history, living situation, social support, travel history, animal exposures, and hobbies. An explanation that drug use, sexual activity, travel to certain areas, or animal exposures may impact future health and future testing if the person becomes ill can be helpful in having a patient be forthcoming with personal information. Obstetrics and gynecologic history including the use of contraception, cervical cancer screening, and the person’s interest in having children in the future should be discussed. Family history, potential for tuberculosis (TB) exposure, previous TB testing and TB treatment history should all be obtained and documented in a standardized manner.

Patients who are establishing care with a new provider, should be offered an opportunity to explore concerns around confidentiality or any other barriers they may have experienced in accessing healthcare services, so that these may be addressed. Information on duration of therapy and reasons for changing treatments or for breaks in therapy are important to understand. A similar history as described above should be obtained. Outside records should be requested and reviewed for information on: ART, other medications, barriers to compliance with ART or medical visits, CD4 counts, genotyping, viral loads, HIV related, and non-HIV-related medical conditions, and immunizations.

A comprehensive physical exam should be part of the initial evaluation and is a time to establish a baseline and to assess for any evidence of HIV-related conditions. Special attention should be paid to the skin, genital and eye examinations which are often cursory during otherwise comprehensive examinations.

Documentation of both a comprehensive history and exam at the time of the first HIV-related visit or shortly after this visit is very helpful when a patient presents later with a new concern or finding. Knowing that the patient had no axillary lymph nodes when seen 6 months ago and now has a 2 cm node and spent a summer in Arizona 6 years ago may allow for the clinician to more quickly consider a likely diagnosis and order appropriate testing.

People newly diagnosed with HIV infection should start on ART as soon as they have an understanding of why ART is recommended and feel ready to be compliant with ART. Studies have shown that people living with HIV are more likely to be successfully retained in care if they start ART at the time of their first visit [3,15]. Rapid ART initiation decreases the chance of transmission to seronegative partners by decreasing the duration of detectable virus [2,11]. Rapid ART also increases the likelihood people living with HIV will maintain viral suppression [15]. Early ART in asymptomatic people living with HIV has been shown to decrease both HIV-related and non-HIV related morbidity and mortality [16,17]. Early ART can prevent compromise of the immune system and potentially decreases the size of the viral reservoir, particularly if started within the first 6 weeks of infection [18]. Most patients with newly diagnosed HIV are candidates for rapid ART initiation, however, clinicians need to ensure there are no reasons to defer rapid ART initiation, which most importantly is absence of concern for cryptococcal or tuberculosis meningitis. Patients who have previously received ART are not usually candidates for rapid ART due to the concern of possible viral resistance.

Treatment-naïve patients with a fully susceptible virus have many once a day options for initial ART which are usually well tolerated. Current initial recommended treatment options for most people living with HIV infection are listed in Table 3 [19]. Recommended baseline laboratory testing is outlined in Table 4 [3,19]. One does not need these results prior to starting ART but the lack of genotyping, HLAB*5701 testing, and hepatitis B testing will affect the ART chosen. Abacavir should not be started without first documenting the patient is HLAB*5701 negative [20] and if the patient is not known to be hepatitis B surface antigen negative a program that includes a Tenofovir formulation combined with Lamivudine or Emtricitabine is recommended. Ideally, renal function should be assessed prior to prescribing TDF. Once initial laboratory testing is available, the ART program started should be reviewed to ensure it is appropriate for the person living with HIV. The Department of Health and Human Services has very detailed guidelines regarding the use of antiretroviral agents in people living with HIV and is a great resource for both the primary care clinician and HIV expert [19].

Following initiation of ART, a viral load should be checked in 2–6 weeks. If the patient is doing well, it should be checked every 4–8 weeks until viral suppression is achieved. Once viral levels fall below the level of detection testing, viral load follow-up can decrease to every 3–4 months, and if the patient stays undetectable for >2 years with a CD4 count >300 cells/mm^3^, viral load testing can decrease to every 6 months and CD4 testing can be done annually. CD4 count monitoring is now optional for people living with HIV who remain virologically suppressed and whose CD4 counts are consistently >500 cells/mm^3^ for at least 2 years [19]. Complete blood count, liver function testing, lipid profile, and renal function should be checked annually in most patients and depending upon the ART and baseline values may need more frequent monitoring [3,21]. Many clinicians check liver function tests, renal function, and lipids 2–4 weeks after ART initiation. Many patients with newly diagnosed HIV infection have opportunistic infections or other HIV-associated conditions that require expert treatment and are beyond the scope of this paper.

Once diagnosed with HIV infection, people are often interested in doing everything possible to improve their immune system. The most important intervention that leads to a long, healthy life for people living with HIV is understanding the importance of adherence to medications and achieving viral suppression. Even patients who do not have optimal improvement in their CD4 count have a low incidence of opportunistic infection if they are able to keep their viral load suppressed for long periods. Primary care providers of persons living with HIV need to ensure that patients are knowledgeable about measures that will help prevent additional infection. This includes antimicrobial prophylaxis when CD4 counts are less than 200 cell/mm^3^, and many other potential behavior changes [1]. Health care providers at times take some of these simple behavioral measures for granted and overlook appropriate counseling. This includes counseling regarding frequent hand washing, avoiding sexual activity that might result in oral exposure to feces, and wearing a mask in crowded situations. Helping patients achieve stable, uncrowded, clean housing will help prevent serious Bartonella infections transmitted by the body louse and ensuring that pets are healthy and free of fleas can also help prevent serious infection. Persons living with HIV need to understand safe sex practices, including how to appropriately use condoms, to avoid acquiring other sexually transmitted infections. Screening for syphilis, gonorrhea, and chlamydia is recommended at least annually for adults living with HIV and more often (every 3–6 months) if their sexual behavior places them at high risk of acquisition. Screening for trichomoniasis is recommended annually for people living with HIV who have receptive vaginal sex [1]. Persons living with HIV also need to understand the importance of not sharing any drug injection equipment and know how to access clean syringes and needles. Annual hepatitis C screening with hepatitis C antibody testing followed by hepatitis C nucleic acid testing for detection of hepatitis C RNA is recommended for all sexually active men who have sex with men, transgender women who have sex with men, and people who inject drugs and are living with HIV. Risk reduction practices to reduce the likelihood of sexually and blood transmitted illnesses should be discussed at each visit. Persons living with HIV who have low CD4 counts should avoid exposure to soil and construction dust as well as bird and bat droppings [1]. If activities that might involve exposure are absolutely necessary, a high-quality form fitting mask should be worn.

Historically, the most common opportunistic infection among people living with HIV in the US has been Pneumocystis pneumonia (PCP) caused by a ubiquitous fungus *Pneumocystis jirovecii.* Most cases of PCP now occur in people who are unaware of their HIV infection or not engaged in care. Adults and adolescents living with HIV should receive primary prophylaxis with Trimethoprim/sulfamethoxazole (TMP/SMX) if their CD4 count is <200 cells/mm^3^ or CD4 <14%. The preferred prophylaxis is one TMP/SMX DS tablet daily, however, TMP/SMX SS once daily and TMP/SMX DS one tablet three times weekly are also effective at preventing PCP. Alternative prophylaxis for patients who cannot tolerate TMP/SMX include dapsone, atovaquone, and inhaled pentamidine. Primary prophylaxis can be discontinued when the viral load is undetectable and the CD4 count is >200 cells/mm^3^ for at least 3 months and in patients who achieve viral suppression for at least 6 months if the CD4 count is >100 cells/mm^3^ [1].

Screening for and prophylaxis against coccidioidomycosis should only be done for people living with HIV infection with CD4 counts < 250 cells/mm^3^ who live or have traveled to an endemic area. Asymptomatic people living with HIV infection, who have a positive coccidioides serology and a CD4 count < 250 cells/mm^3^ should ideally be treated with Fluconazole 400 mg daily until the CD4 count increases to >250 cells/mm^3^ and viral suppression is achieved [1]. Screening with a serum cryptococcal antigen test (CrAg) for cryptococcal disease is recommended in asymptomatic people living with HIV and a CD4 count < 100 cells/mm^3^. A positive test should prompt a careful history and exam to look for clues of cryptococcal disease and a CSF evaluation for CNS infection. Cryptococcal meningitis is one of the few times when initiation of ART should be delayed. Treatment of cryptococcal infection is beyond the scope of this paper and even asymptomatic patients with a high CrAg should be treated by an expert with consolidation therapy as if they have meningitis. People living with HIV with a low CrAg who are asymptomatic or have mild pulmonary disease can be treated with Fluconazole 400–800 mg for 10 weeks followed by 200 mg for a total treatment course of 6 months. Treatment can be discontinued once the CD4 is >100 cells/mm^3^ and viral load is suppressed at 6 months. Maintenance therapy should be resumed for all people living with HIV and a history of cryptococcosis if the CD4 count drops below 100 cells/mm^3^ [1].

Antifungal prophylaxis with itraconazole to prevent histoplasmosis is not generally recommended in the US. Prophylaxis can be considered with Itraconazole 200 mg daily if the person lives in a hyperendemic area with a very high rate of histoplasmosis and has a CD4 count < 150 cells/mm^3^ [1].

Mycobacterium avium complex (MAC) disease is very common among people living with HIV and profound immunosuppression (CD4 < 50 cells/mm^3^). Primary prophylaxis with a macrolide is no longer recommended against MAC if one is able to start ART. If ART is not able to be promptly initiated or if a person cannot obtain viral suppression and has a persistently low CD4 count < 50 cells/mm^3^, then prophylaxis with either clarithromycin or azithromycin is recommended [1].

All people living with HIV should be screened for tuberculosis (TB) at the time of their initial diagnosis with either a tuberculin skin test (TST) or with an interferon gamma release assay (IGRA). Screening tests for TB can be falsely negative in persons living with HIV and low CD4 counts. An IGRA may be more sensitive in people living with HIV and will not be falsely positive in people who have previously received the Bacille Calmette-Gueri (BCG) vaccine. False positive TSTs are common in people previously immunized with BCG. All persons living with HIV also need to be carefully examined for active TB regardless of the result of their screening test. If the TST is >5 mm or an IGRA is positive, the person living with HIV should be considered to have a positive screening test for TB. Once the provider is certain that the person does not have active TB, treatment for latent TB should be started. The preferred latent TB program for persons living with HIV depends on their ART program, comorbidities, and social history, including ability to avoid alcohol. If Isoniazid (INH) alone is used, then pyridoxine supplementation (to decrease the chance of neuropathy) and a 9-month course is recommended. Other effective programs for people living with HIV include a 4-month course of rifampin or 12 weeks of once weekly Rifapentine and INH administered by directly observed therapy. Rifampin alone and INH/Rifapentine have a higher chance of successful completion since they are shorter than the INH program alone; however, careful attention to ART and other drug–drug interactions is necessary due to the inclusion of a rifamycin. Screening for latent TB should occur annually for people living with HIV who are at risk of exposure [1].

Toxoplasmosis is a common parasite that is acquired primarily by eating undercooked meat containing tissue cysts or ingesting oocysts shed in cat feces that have sporulated in the environment. Approximately 10% of people in the US are seropositive for Toxoplasmosis and at risk of reactivation with cellular immunosuppression. The seroprevalence is much higher in some European, Latin American, and African countries. The most common clinical syndrome of reactivation is toxoplasma encephalitis (TE) in people with AIDS. People living with HIV who have CD4 counts <100 cells/mm^3^ and are seropositive for *Toxoplasma gondii* should be started on prophylaxis. These people also should be receiving prophylaxis as described above for PCP. Fortunately, the preferred prophylaxis for PCP, TMP/SMX DS once daily is effective at preventing TE. TMP/SMX DS three times weekly and atovaquone are also effective at preventing TE. Prophylaxis for TE can be discontinued when the CD4 count is >200 cells/mm^3^ for at least 3 months and can be discontinued if the viral load is suppressed for at least 3 months and expected to stay suppressed with a CD4 count >100 cells/mm^3^ [1].

Immunizations are an important part of primary care and an important tool for keeping people living with HIV healthy. The 13-valent pneumococcal conjugate vaccine (PCV13) should be given to all adults and adolescents living with HIV who have never received any pneumococcal vaccine. Those with CD4 >200 cells/mm^3^ should receive the 23-valent pneumococcal polysaccharide vaccine (PPSV23) 8 weeks or more post the PCV13 vaccine. Those with CD4 counts <200 cells/mm^3^ can be given the PPSV23 vaccine 8 weeks after the PCV13 but delaying until after the CD4 count increases to >200 cells/mm^3^ is recommended if there is good reason to believe the CD4 count is likely to significantly improve with ART. This allows for a more robust immune response to the vaccine. The PPSV23 should not be delayed indefinitely while waiting for CD4 counts to improve in patients who do not have good immune recovery or who are poorly adherent to ART. Adults who have already received a PPSV23 should be given a PCV13 if it has been at least 1 year since the PPSV23. A repeat PPSV23 5 years after the first dose is recommended and a third dose after the age of 65 if it has been 5 years or longer since the last dose. No more than three lifetime doses of PPSV23 are recommended. [1]

Adults living with HIV should receive a two-dose series at least 8 weeks apart of a meningococcal A, C, Y, and W serogroup vaccine and revaccination every 5 years for life is recommended. Meningococcal B serogroup vaccine is not routinely recommended but can be considered for young adults living in group settings such as the general population [1].

The inactivated influenza vaccine is recommended annually for all persons living with HIV. The live-attenuated influenza vaccine is contraindicated. Tetanus, diphtherial, and pertussis vaccine recommendations are the same as for the general population. All people living with HIV should receive a COVID-19 vaccine series, and the three-dose primary series of a mRNA vaccine is recommended for patients with advanced HIV (CD4 < 200 cells/mm^3^ or <14%) followed by a 4th booster dose at least 3 months after the third dose. All persons living with HIV who have CD4 counts ≥ 200 cells/mm^3^ should have a booster dose 6 months after completing the mRNA two-dose series. If patients received the Johnson and Johnson COVID-19 vaccine, then a second dose ideally with a mRNA COVID-19 vaccine at least 4 weeks after the first dose and a booster dose of a mRNA vaccine at least 2 months after the 2nd dose if CD4 < 200 cells/mm^3^ is recommended. COVID-19 vaccination guidelines are rapidly changing, and readers are encouraged to review updated guidelines from the CDC [22].

The measles, mumps and rubella vaccine (MMR) is a live virus vaccine and should be given to unvaccinated adults with a CD4 > 200 cells/mm^3^ who were born in the US after 1956 or have immigrated to the US and have no evidence of immunity to MMR. The MMR vaccine is contraindicated in people living with HIV who have CD4 counts < 200 cells/mm^3^ or who are pregnant. Pregnancy should be delayed for at least 4 weeks after receiving the MMR. The MMR series is two doses at least one month apart [3].

People living with HIV who have no history of varicella or zoster and who have not been previously immunized against varicella should have serology against varicella assessed. People living with HIV who are varicella non-immune and have CD4 count > 200 cells/mm^3^ should be given two doses of the live attenuated varicella vaccine (Varivax) 3 months or more apart. Varivax is a contraindicated if CD4 < 200 cells/mm^3^ and in pregnancy [1]. An inactivated recombinant varicella vaccine (RZV), trade name Shingrix, is recommended to prevent reactivation of varicella/zoster in all adults over 50 years and in October of 2021 was recommended by the Advisory Committee on Immunization Practices for all adults 19 years and older who are immunosuppressed and at increased risk for varicella/zoster reactivation [23]. All adults living with HIV who are at risk of shingles should be vaccinated with RZV.

Hepatitis A vaccination is recommended for everyone who is nonimmune and especially for all men and transgender women who have sex with men, people who use injection drugs, people with underlying liver disease, and anyone who is at risk for hepatitis A and not immune. Hepatitis B vaccination is also recommended for all people living with HIV who are not infected or immune. Both hepatitis A and B vaccine immune responses are reduced in patients with low CD4 cell counts (<200 cells/mm^3^) and detectable viral loads. The decision to vaccinate shortly after diagnosis or delay until viral load is suppressed and CD4 count has increased needs to be weighed against the risk of infection. Persons who fail to respond to either vaccine should be revaccinated ideally after immune reconstitution [1].

All people living with HIV between the ages of 9 and 26 should be vaccinated against HPV with a 3-dose series if they have not completed the HPV vaccine series prior to diagnosis. People aged 27–45 living with HIV should be considered for the HPV vaccine, but little data on efficacy for people living with HIV in this age group is available [1].

Many primary care providers become involved in the care of persons living with HIV once they are on stable ART and doing well from the HIV standpoint. Primary care of these patients is very similar to the general population; however, liver disease, coronary artery disease, HPV-related cancers, osteoporosis, and mental health concerns are more common among people living with HIV. Cancer screening for colon, breast, lung, and prostate cancer is similar to the general population and United States Preventative Task Force (USPTF) recommendations apply to persons living with HIV. Cirrhosis and HBV infection are more common in the US in people living with HIV than the general population. Screening for hepatocellular cancer with a liver ultrasound every 6 months is recommended for people with cirrhosis. Screening for hepatocellular cancer is also recommended for people infected with HBV and active hepatitis (e.g., elevated serum ALT) and/or high viral load (i.e., >100,000 copies/mL (20,000 international units/mL)), with a family history of hepatocellular cancer, for Asian males over the age of 40, Asian females over the age of 50, and for all those of African descent [24].

HPV-related cancers are much more common in persons living with HIV, and all persons with a cervix who are between 21 and 30 should have a cervical Pap smear at the time of diagnosis and, if normal, repeated annually. If three consecutive annual Pap smears are normal, the frequency of screening can decrease to every 3 years. People over 30 have the option of following the above recommendations or having both a Pap and HPV testing and if both are negative, Pap with HPV testing can be repeated every 3 years without the need for three consecutive annual Pap smears. Unlike the general population, screening should continue past the age of 65 and when to stop screening should be individualized depending upon the person’s risk of acquiring new HPV and their other comorbidities. All patients with an abnormal cervical Pap or + HPV test for HPV genotype 16 or 18 should be referred for a colposcopy. Screening for anal cancer is recommended for all persons living with HIV and genital warts if appropriate follow-up including high resolution anoscopy is available [3]. Most HIV experts recommend anal cancer screening with an anal Pap for all men and transgender women who have sex with men, and many recommend screening for anal cancer for all adults living with HIV. There is no guideline or consensus on who should be screened and how often screening should occur. Screening for anal cancer should only occur if referral for high resolution anoscopy with biopsy if indicated and ablative treatment is available [1,3].

The aging of the population living with HIV in the US has come with the realization that many conditions and frailty associated with aging occur more commonly and often earlier in people living with HIV compared to the general population. [25] Clinical features of frailty include self-reported weight loss of >5%, self-reported exhaustion, low level of physical activity, decreased 4 m walk speed, and poor grip strength. Personalized interventions aimed at maximizing health and improving quality of life are indicated when frailty is found. A formal physical activity program with resistance training to combat sarcopenia, addressing modifiable causes of fatigue, nutritional supplementation if weight loss, and vitamin D supplementation if vitamin D deficient are recommended [21]. Depression and other comorbidities are common in frail individuals living with HIV and need to be addressed [25].

Polypharmacy and drug–drug interactions between ART, non-HIV related medications, and chemotherapy become increasingly common as people living with HIV age. Pharmacists with ART expertise are important members of the multi-disciplinary team caring for people living with HIV and can help ensure that people living with HIV remain virologically suppressed even when their medication programs become increasingly complex.

People living with HIV have been shown to have an increased risk of cardiac disease, even when traditional cardiac risk factors are controlled for [26]. The impact of ART on cardiovascular disease (CVD) has varied across studies. Suppressive ART does not appear to completely decrease the risk of a major cardiovascular event to that of the general population and concerns have been raised that ART is contributing to the risk of CVD. Interrupting ART has, however, been shown in a classic study to significantly increase the risk of both fatal and nonfatal myocardial infarction [27]. Many HIV experts believe that chronic inflammation from viremia prior to the diagnosis of HIV increases the risk of CVD, bone loss, and liver disease. People who have advanced HIV at the time of their diagnosis or who delay starting ART can be expected to experience more of these non-HIV-related comorbidities. Primary care providers of people living with HIV need to be comfortable with motivational interviewing to help patients change behaviors that increase their risk of CVD (smoking, dietary changes, and exercise) and need to be comfortable aggressively managing traditional risk factors: hypertension, hyperlipidemia, and diabetes. Blood pressure and weight should be checked at the time of each visit and addressed when appropriate. Patients and providers need to realize that once a person is adherent and well-controlled with ART, often behavior changes—particularly quitting smoking—is the intervention that is likely to have the most beneficial effect on their long-term health.

Cardiovascular risk calculators were not created for estimating coronary artery disease (CAD) risk in a population living with HIV and likely underestimate the risk of a CAD event for many people living with HIV. Despite this, the American Heart Association/American College of Cardiology (AHA/ACC) Pooled Cohort Equations CV Risk Calculator (PCE), is a reasonable starting place and if elevated a provider can be confident that the person is at increased risk of CAD. Behavior changes that will decrease the person’s risk of CAD should be encouraged and discussed at every routine visit. Lipids should be checked at the time of diagnosis and rechecked after on a stable ART program and following changes in ART. ART programs vary in their effect on lipids. Lipids should be rechecked at least every 5 years, with more frequent monitoring if abnormal or other CVD risk factors are present. Hyperlipidemia management with statin therapy is similar to the general population with the caveat that some ART and statins cannot be used together, and close attention needs to be paid to drug–drug interactions.

Diabetes is also seen more commonly in persons living with HIV than the general population. Screening with a glucose or HbA1c should occur at the time of diagnosis and, if normal, a glucose should be checked 1–3 months after ART and every 3–12 months depending upon the patient’s individual situation. Glucose is recommended for screening for diabetes for patients on ART since the HbA1c may underestimate fasting glucose in people on ART [28]. A fasting blood sugar of ≥ 126 mg/dL is diagnostic of diabetes. Once diabetes is diagnosed persons living with HIV should have regular monitoring of their HbA1c and correlation with their blood glucose should be done to ensure sugars are well-controlled. People living with HIV may also have an increased risk of hypertension and should be screened for hypertension at the time of every visit and managed similar to the general population.

Fractures are more common among persons living with HIV than a matched population of uninfected persons. This increased risk is not purely due to low bone density, but low bone density is a modifiable risk factor for fracture. Screening for osteopenia and osteoporosis is recommended for men older than 50 years, for all postmenopausal women, and for anyone with a fragility fracture living with HIV [3]. The optimal interval for rescreening people living with HIV is unknown, but it is reasonable to base the interval on baseline aBMD, similar to the general population. The management of osteopenia and osteoporosis in people living with HIV is currently similar to the HIV uninfected population. ART that achieves viral suppression has been shown to decrease fracture risk and leads to normalization of the bone-remodeling process during highly active ART [29,30]. ART programs containing TDF should ideally be modified in patients with osteopenia or osteoporosis, and often the simplest and safest change is to substitute TAF for TDF.

Neurocognitive disorders are reported to be more common in people living with HIV, but severe HIV-associated dementia is much less common in the potent ART era [31]. HIV dementia often responds to ART but not everyone notes a complete return to normal cognition. A large study of over 15,000 people living with HIV found that lower CD4 counts, older age at seroconversion, longer duration of HIV, and the presence of a prior AIDS defining diagnosis were risk factors for development of HIV-associated dementia [32]. Screening for deficits by inquiring about memory challenges, reasoning, and attention with more formal testing if the person living with HIV or those closest to them have noted symptoms is a reasonable approach. Some studies have suggested that the min-mental status exam is an insensitive tool for detection of HIV associated neurological disorder and the Montreal Cognitive Assessment may be a better bedside test [33,34].

Mental health and substance use disorders are common among people living with HIV. Depression has been found to be a major risk factor for HIV acquisition and can lead to poor adherence to clinic visits and ART [35]. Screening for substance abuse and for depression should be done at the time of initial diagnosis and at least annually. An important role of the primary care provider of people living with HIV is ensuring these disorders are identified and treated. People living with HIV who also suffer from depression and addiction can easily overwhelm clinicians with the complexity of their situation. Primary care providers should be knowledgeable regarding the resources available in their community and be ready to link people to substance abuse treatment, mental health providers, social workers, and others who can help with housing and other critical needs. Primary care clinicians who are able to link people living with HIV to appropriate treatment and offer an ongoing supportive therapeutic relationship are often the key to long-term health for these complex individuals [3,35].

## Figures and Tables

**Table 1:** Clues to chronic HIV Infection.

Active tuberculosis Herpes zoster in a healthy person younger than 50 y New severe psoriasis or other new unexplained severe skin disorder History of hepatitis B or C Cervical cancer Thrush not related to recent antibiotic use Unexplained cachexia or weight loss Diffuse lymphadenopathy Unexplained thrombocytopenia, leukopenia, or anemia History of an opportunistic or unusual infection in an otherwise healthy individual Prolonged unexplained illness despite evaluation Any history of sexually transmitted infection Patients with long-standing/chronic HIV are often asymptomatic Adapted from Kasten MJ. Human immunodeficiency virus: the initial physician-patient encounter. Mayo Clin Proc. 2002;77(9):957–962; quiz 962–963.

**Table 2:** Clues to acute HIV Infection.

Most but not all patients have fever Unexplained viral like illness (combination of any of the following: fever, pharyngitis, fatigue, rash, lymphadenopathy, diarrhea, myalgia, elevated LFTs, atypical lymphocytosis) New night sweats, or weight loss Aseptic meningitis, or encephalitis Acute psychiatric disorder New thrombocytopenia Opportunistic infection or unusual infection in an otherwise healthy person

**Table 3:** Recommended ART for most treatment-naïve patients with no known exposure to resistant virus [19].

Bictegravir/tenofovir alafenamide/emtricitabine (co-formulated tablet) Dolutegravir/abacavir/lamivudine (co-formulated tablet)—only for individuals who are HLA-B*5701 negative and without chronic hepatitis B virus (HBV) coinfection Dolutegravir plus (emtricitabine or lamivudine) plus (tenofovir alafenamide (TAF) or tenofovir disoproxil fumarate (TDF)) Dolutegravir/lamivudine (co-formulated tablet) (AI)—except for individuals with HIV RNA >500,000 copies/mL, HBV coinfection, or when ART is to be started before the results of HBV testing or HIV genotypic resistance testing for nucleoside reverse transcriptase mutations are available

Abbreviations: antiretroviral therapy (ART); hepatitis B virus (HBV).

**Table 4:** Recommended laboratory testing at time of initial evaluation [3,19].

HIV antigen/antibody testing if written evidence of diagnosis not available or if viral load is low or undetectable CD4 cell count and percentage Plasma HIV RNA (HIV viral load) Baseline genotypic resistance testing (integrase resistance testing is not needed unless patient has previously been on an integrase inhibitor) HLAB*5701 if use of abacavir is being considered Complete blood cell count with differential Alanine aminotransferase, aspartate aminotransferase, total bilirubin, alkaline phosphatase Electrolytes, blood urea nitrogen, creatinine Lipid profile and blood glucose Urinalysis Gonorrhea and chlamydia nucleic acid amplification testing with sites based on potential exposure (e.g., urine, vaginal, rectal, oropharyngeal) Trichomoniasis testing in all persons who have vaginal sex Syphilis testing using local protocol (either rapid plasma regain or treponemal-specific antibody tests) Latent *Mycobacterium tuberculosis* testing (tuberculin skin test or IGRA; IGRA preferred if history of BCG vaccination) Varicella virus: anti-varicella IgG if no known history of vaccination, chicken pox, or shingles Viral hepatitis A, B, and C testing: HBsAg, HBsAb, HBcAb, HCV antibody, and if positive HCV NAT for HCV RNA; HAV total or IgG antibody Measles titer if not born before 1957 in the US, and no written documentation of adequate MMR vaccination, or serologic evidence of immunity. Persons born in the 1960s may have been vaccinated with a vaccine other than MMR and have waning immunity. Patients may opt to receive a booster MMR vaccine rather than checking serology. Cytology: cervical Papanicolaou (Pap) test for all people living with HIV who have a cervix; and consider anal Pap test for all people living with HIV Glucose-6-phosphate dehydrogenase screen for deficiency in appropriate racial or ethnic groups Pregnancy test in persons of childbearing potential Coccidioidomycosis serology for those living in endemic areas with CD4 <250 cells/mm^3^ **Optional Testing to Consider** Serum cryptococcal antigen for persons with CD4 cell count <100/mm^3^ Serum testosterone level in cisgender males with fatigue, weight loss, loss of libido, erectile dysfunction, or depression Chest radiography

Abbreviations: Bacillus Calmette–Guerin (BCG); hepatitis A virus (HAV); HBcAb, HBsAb, hepatitis B core antibody (HBcAb), (HBsAb); hepatitis B surface antigen (HBsAg); hepatitis C virus (HCV); human immunodeficiency virus (HIV); immunoglobulin G (IgG); interferon-γ release assay (IGRA); measles mumps rubella (MMR), nucleic acid test (NAT). Adapted from Melanie A. Thompson, 1,a Michael A. Horberg, 2,a Allison L. Agwu, 3 Jonathan A. Colasanti, 4 Mamta K. Jain, 5 William R. Short, 6 Tulika Singh, 7 and Judith A. Aberg 8. Primary Care Guidance for Persons With Human Immunodeficiency Virus: 2020 Update by the HIV Medicine Association of the Infectious Diseases Society of America. CID Nov., https://doi.org/10.1093/cid/ciaa139 (accessed on 15 March 2022).

## Data Availability

Not applicable.
